# Acidic pH Decreases the Endonuclease Activity of Initiator RepB and Increases the Stability of the Covalent RepB-DNA Intermediate while Has Only a Limited Effect on the Replication of Plasmid pMV158 in *Lactococcus lactis*

**DOI:** 10.3389/fmolb.2021.634461

**Published:** 2021-03-05

**Authors:** Rafael Valdelvira, Lorena Bordanaba-Ruiseco, Cristina Martín-Huestamendía, José Angel Ruiz-Masó, Gloria del Solar

**Affiliations:** Department of Microbial and Plant Biotechnology, Centro de Investigaciones Biológicas, Consejo Superior de Investigaciones Científicas, Madrid, Spain

**Keywords:** rolling circle replication (RCR), promiscuous plasmid pMV158, nucleophilic attack, endonuclease activity, strand-transfer activity, covalent RepB-DNA adduct

## Abstract

Plasmid vectors constitute a valuable tool for homologous and heterologous gene expression, for characterization of promoter and regulatory regions, and for genetic manipulation and labeling of bacteria. During the last years, a series of vectors based on promiscuous replicons of the pMV158 family have been developed for their employment in a variety of Gram-positive bacteria and proved to be useful for all above applications in lactic acid bacteria. A proper use of the plasmid vectors requires detailed knowledge of their main replicative features under the changing growth conditions of the studied bacteria, such as the acidification of the culture medium by lactic acid production. Initiation of pMV158 rolling-circle replication is catalyzed by the plasmid-encoded RepB protein, which performs a sequence-specific cleavage on one of the parental DNA strands and, as demonstrated in this work, establishes a covalent bond with the 5′-P end generated in the DNA. This covalent adduct must last until the leading-strand termination stage, where a new cleavage on the regenerated nick site and a subsequent strand-transfer reaction result in rejoining of the ends of the cleaved parental strand, whereas hydrolysis of the newly-generated adduct would release the protein from a nicked double-stranded DNA plasmid form. We have analyzed here the effect of pH on the different *in vitro* reactions catalyzed by RepB and on the *in vivo* replication ability of plasmid pMV158. We show that acidic pH greatly impairs the catalytic activity of the protein and reduces hydrolysis of the covalent RepB-DNA adduct, as expected for the nucleophilic nature of these reactions. Conversely, the ability of pMV158 to replicate *in vivo*, as monitored by the copy number and segregational stability of the plasmid in *Lactococcus lactis*, remains almost intact at extracellular pHs ranging from 7.0 to 5.0, and a significant reduction (by ∼50%) in the plasmid copy number per chromosome equivalent is only observed at pH 4.5. Moreover, the RepB to pMV158 molar ratio is increased at pH 4.5, suggesting the existence of compensatory mechanisms that operate *in vivo* to allow pMV158 replication at pH values that severely disturb the catalytic activity of the initiator protein.

## Introduction

Rolling-circle replication (RCR) was first described for single-stranded DNA (ssDNA) coliphages about half a century ago (Gilbert and Dressler, 1968; Dressler, 1970; Baas, 1985). Later, the RCR mechanism was reported for various small multicopy plasmids isolated from Gram-positive (Koepsel et al., 1985; te Riele et al., 1986; del Solar et al., 1987) and Gram-negative (Yasukawa et al., 1991; Solar et al., 1993) bacteria, as well as from archaea (Marsin and Forterre, 1999) and mitochondria (Backert et al., 1997; see also reviews by Khan, 2005; Ruiz-Masó et al., 2015). Recently, evidence of RCR has been obtained for some excised integrative and conjugative elements (ICE) (Burrus, 2017), and this replicative mechanism may also account for the maintenance of certain integrative and mobilizable elements (IME) while they are transiently excised from the chromosome (Guédon et al., 2017). Also ssDNA viruses of plants and animals are known to replicate by a RCR-like mechanism (Dolja and Koonin, 2011; Chandler et al., 2013). Basically, RCR initiation implies the sequence-specific cleavage of the non-template strand of a double-stranded DNA (dsDNA) form of the genetic element to be replicated. The cleavage reaction, which is catalyzed by a Rep protein encoded by the genetic element, generates a 5′-phosphate end covalently attached to the Rep protein (the so-called Rep-DNA adduct), and a free 3′-OH end that serves as a primer for the synthesis of the DNA using host cell enzymes. Hence, a singularity of the RCR is that the newly-synthesized DNA remains covalently linked to the cleaved parental strand during the entire elongation step. In all reported cases, a Tyr residue located in a conserved motif of the Rep protein is used as the nucleophile for the initial endonucleolytic cleavage of the parental DNA (Koonin and Ilyina, 1993; Chandler et al., 2013).

RCR in plasmids is subjected to an strict control that allows them to maintain their characteristic intracellular concentration (i.e., copy number) in a given host under defined growth conditions, thus reducing plasmid loss and metabolic burdening of the cell. Control of plasmid RCR is exerted by 1) self-encoded elements that inhibit the synthesis of the Rep initiator protein at the transcriptional and/or translational level, and 2) complementary mechanisms that avoid recycling and ensure inactivation of the Rep protein, so that each initiator molecule drives a single round of replication (reviewed in Ruiz-Masó et al., 2015).

Catalytic residues involved in the RCR initiation and termination of the leading strand have been best characterized in Rep proteins of the pT181 and pC194 plasmid families. The initiation cleavage at the specific nick site of one of the parental DNA strands is catalyzed by a Tyr residue located either in one of the subunits of a dimeric Rep protein (pT181 plasmid family) or in a monomeric Rep protein (pC194 plasmid family). In the members of the pT181 family, once the synthesis of the leading-strand has been completed, the catalytic Tyr of the second Rep subunit mediates a cleavage reaction on the reconstituted nick sequence and becomes covalently attached to the 5′-end of the newly-synthesized strand. On its turn, the 3′-OH end generated in this reaction acts as a nucleophile that attacks the phosphotyrosine bond of the first Rep subunit and thus leads to the generation of a circular ssDNA intermediate corresponding to the parental strand cleaved at the replication initiation step, which will serve as the template for the lagging-strand synthesis. The released catalytic Tyr of the first Rep subunit still mediates one more cleavage reaction on a second nick sequence that has been reconstituted by extension of the nascent leading strand by ∼10 additional nucleotides beyond the nick site, and becomes covalently attached to this short oligonucleotide (Khan, 2005). This termination mechanism has two important implications for the regulation of the number of replication rounds driven by the same Rep dimer: 1) it prevents recycling (i.e., continuous rounds of leading-strand synthesis driven by the same Rep molecule), and 2) the heterodimeric Rep/Rep-DNA protein is inactive in replication initiation (Novick, 1998; Ruiz-Masó et al., 2015). In contrast, in plasmids of the pC194 family, a Glu residue of the monomeric Rep protein mediates the hydrolysis of the DNA at the specific site of the reconstituted nick sequence during the replication termination step. Hence, covalent linkage of Rep to the 5′-phosphate end of the newly-synthesized leading strand and subsequent recycling of the protein are prevented (Noirot-Gros et al., 1994). The 3′-OH end generated in the cleavage of the reconstituted nick sequence next attacks the adduct, thus releasing the free protein along with the circular ssDNA intermediate.

RCR plasmids of the pMV158 family are singular in that their Rep protein rather resembles the Rep initiators of small eukaryotic viruses: all of them form hexameric rings with a central electropositive channel that can lodge a ssDNA but not a dsDNA fragment (Enemark and Joshua-Tor, 2006; Boer et al., 2009, 2016). The atomic structure of the full-length RepB initiator encoded by pMV158 has been solved and remains to date the only published example among the Rep proteins from RCR plasmids and bacteriophages (Boer et al., 2009). The crystalographic analysis of RepB shows a hexameric molecule whose protomers consist of two domains, namely the N-terminal origin binding domain (OBD) and the C-terminal oligomerization domain (OD), conected by a short hinge region that allows multiple orientations of the OBDs relative to the rigid cylindrical scaffold constituted by the ODs. The OBD accounts for both the specific dsDNA binding ability and the catalytic activities of the protein, whereas the OD is responsible for hexamerization (Boer et al., 2009). The OBD and OD domains of RepB have been purified separately as monomeric and hexameric proteins, respectively, and the OBD was shown to retain the DNA binding and catalytic activities of the protein (Boer et al., 2016). The RepB OBD domain belongs to the Rep class of the HUH endonuclease superfamily, which is featured by a His-hydrophobic-His (HUH) sequence motif involved in the coordination of the divalent metal ion of the active site (Chandler et al., 2013; Boer et al., 2016).

Although the mechanistic details of the reactions that take place in initiation and termination of the leading-strand synthesis of pMV158 (and related plasmids) remain still unveiled, they have to involve the initial cleavage of the parental strand at the nick site and the formation of a covalent adduct, most likely mediated by the Tyr99 residue located at the active center of the RepB protein (Boer et al., 2009). This adduct must last until the termination step, despite its *in vitro* identification has remained elusive for a long time (Moscoso et al., 1995; Moscoso et al., 1997). On the other hand, termination must involve at least a new cleavage reaction, catalyzed either by Tyr99 of a different protein subunit or by a second catalytic residue of the same subunit, as well as a strand-transfer reaction that seals the cleaved parental strand leading to the generation of the ssDNA replicative intermediate (del Solar et al., 1987). Both cleavage and strand-transfer reactions imply nucleophilic attacks, and hence could be expected to be sensitive to acidity, although very few studies have been performed about the effect of the pH on these reactions (Lovendahl et al., 2017). Moreover, despite the lack of information about the stability of Rep-DNA covalent adducts involving RCR initiators, the phosphoryl bond of phosphotyrosine residues of various proteins has been shown to be quite stable to basic conditions that give complete hydrolysis of peptide bonds (Martensen, 1982), and the phosphotyrosine linkage between *Escherichia coli* or *Micrococcus luteus* DNA topoisomerase I is stable in aqueous buffers ranging from pH 1 to 13 (Tse et al., 1980).

In this work, we have taken advantage of the great promiscuity of plasmid pMV158, which has been shown to replicate in the γ-proteobacterium *E. coli*, in several actinobacteria, and in many species from the Firmicutes phylum, including lactic acid bacteria (LAB) that can grow under very low pH conditions. Whereas the intracellular pH (pH_i_) of neutralophilic hosts (like *E. coli*) is maintained close to neutral when the medium is acidified, many LAB have been reported to reduce their pH_i_ as the extracellular pH (pH_ex_) decreases, thus generating smaller proton gradients across the membrane (Siegumfeldt et al., 2000). Studies on the homeostasis against acid stress in *Lactococcus lactis* subsp. *cremoris* MG1363 have revealed the relationship between the pH of the culture medium and the actual value of the pH_i_ in this model LAB strain (Mercade et al., 2000). This information has allowed us to test the effect of the pH not only on the *in vitro* reactions of cleavage, strand transfer and hydrolysis of the adduct, but also on the *in vivo* replication of pMV158 by checking its copy number and segregational stability in the lactococcal host.

## Materials and Methods

### Bacterial Strains and Growth Conditions

*L. lactis* subsp. *cremoris*, strain MG1363 (Gasson, 1983) harboring plasmid pMV158 (*L. lactis* MG1363/pMV158) was cultivated routinely in M17 (Difco™) medium supplemented with yeast extract to a final concentration of 0.5% and with 0.5% glucose (M17′; Sánchez et al., 2008) at 30°C without aeration. When needed, tetracycline (Tc) was added at a concentration of 1 μg/ml. Growth was monitored with the aid of a Spectronic 20D+ equipment by checking the optical density (OD) at 660 nm.

For growing *L. lactis* MG1363/pMV158 at different pH conditions, the pH of the M17′ medium was adjusted with HCl to the values analyzed in this study: 7.0, 6.5, 6.0, 5.5, 5.0, and 4.5. A bacterial inoculum was prepared in M17 supplemented with 0.5% glucose and 1 μg/ml Tc, and grown statically at 30°C to an OD_660_ of 0.4. Several aliquots of this starter culture were taken, and the cells washed and resuspended in Tc-free M17′ adjusted to different pHs, to obtain cultures at an initial OD_660_ of 0.1. These cultures were incubated under the same conditions until the OD_660_ reached again 0.4, which corresponds to two generations of cellular growth. This procedure of cell collection, washing, dilution in antibiotic-free M17′ adjusted to the corresponding pH and incubation until OD_660_ of 0.4 was repeated for 20 generations of cellular growth, thus avoiding an excessive culture medium acidification due to the production of lactic acid by the bacteria. In fact, by following this procedure we only observed decreases of less than 0.3 units of pH in the cultures at OD_660_ of 0.4 respect to the initial pH value of the medium. Aliquots corresponding to 10 and 20 generations of cell growth at the different pHs were taken with two purposes: 1) extraction of genomic DNA (gDNA) for plasmid copy number (PCN) determination, and 2) analysis of the segregational stability of the plasmid by determination of the percentage of colony forming units (cfu) resistant to Tc (Tc^R^). To estimate the percentage of Tc^R^ cfu, appropriate dilutions of culture samples were spread on M17-agar plates supplemented with glucose 0.5% and containing or not Tc.

### Genomic DNA Preparations

Lactococcal gDNA, used as template for real-time quantitative PCR (qPCR), was isolated as described in (Garay-Novillo et al., 2019). Purified gDNA was digested with EcoRI, which linearizes the pMV158 plasmid DNA but leaves intact the template DNA segments containing the plasmidic and chromosomal amplicons that will be amplified in the qPCR assays. This method was developed to obtain accurate qPCR-based copy number results for plasmids (Providenti et al., 2006). Concentration of the gDNA was determined with a Qubit fluorometer by using the Qubit HS dsDNA Assay Kit (Molecular Probes). DNA integrity was examined by 0.8% agarose gel electrophoresis and DNA staining with GelRed (Biotium). Irrespective of the pH of the medium, the gDNA yield was ∼10 µg from 1 ml of culture at OD_660_ of 0.8. Hence, similar intracellular concentrations of chromosomal DNA were inferred for lactococcal cells grown at the different pHs.

### Determination of PCN in *L. lactis* by qPCR and Agarose Gel Quantification

For the determination of the PCN per chromosomal equivalent by qPCR, two primer pair sets specific to the PcrA helicase single-copy reference gene (*pcrA*) of *L. lactis* MG1363 (Wegmann et al., 2007) and to the RepB replication protein gene (*repB*) of pMV158 were used (Garay-Novillo et al., 2019). Criteria used during primer design were that primers had a predicted Tm of ∼59°C and that they generated amplicons of ∼140 bp in length. qPCRs were conducted as described (Ruiz-Masó et al., 2017). Diluted preparations of EcoRI (Nzytech)-digested gDNA (5, 1.7, and 0.17 ng per reaction) were analyzed using 0.5 µM of the specific forward and reverse primers of the primer pair used. Three independent qPCR trials were conducted for each template source. In each trial, triplicate samples of the three different amounts of template were analyzed. PCN of pMV158 at the different pH conditions studied was calculated as described (Garay-Novillo et al., 2019).

For PCN determination by agarose gel quantification, gDNAs samples were loaded into 0.8% agarose gels, run for 60 min at 6.5 V/cm and dyed with GelRed for 10 min. Gels were visualized with the aid of a Gel Doc (Bio-Rad). Non-saturated images of the gels were analyzed with the Quantity One v4.5.2 software (Bio-Rad). Plasmid bands corresponding to the covalently-closed circular (CCC) and open circular (OC) forms and chromosomal DNA band in each lane were identified and their densities analyzed. For estimation of the pMV158 PCN in cells grown at different pHs, the ratio of the signal of plasmid (CCC × 1.36 + OC; Projan et al., 1983) to the signal of chromosome was quantitatively compared with that of *L. lactis*/pMV158 cells grown at pH 7.0, whose value was taken as a reference to which the PCN determined by qPCR was assigned.

### Protein Purification

Hexameric full-length RepB (RepB_6_) and His-tagged OBD (hereafter termed OBD) were purified as described previously (Ruiz-Masó et al., 2004; Boer et al., 2009). Protein concentration was measured by two methods: 1) spectrophotometrically using the theoretical molar absorption coefficient, and 2) SDS-PAGE and quantitative densitometry. In the latter method, gels were stained with Coomassie Brilliant blue and the densitometric value of the specific protein band was quantitatively compared with that of a RepB_6_ or OBD sample of known concentration.

### Identification of the RepB-DNA Covalent Adduct

The formation of a covalent complex between RepB and the 5′-phosphate end of the cleaved DNA substrate was analyzed by using RepB_6_ and His-tagged OBD proteins. For the detection assays, 5 pmol of the 27-mer oligo substrate (5′-TGC​TTC​CGT​ACT​ACG/ACC​CCC​CAT​TAA-3′; where “/” indicates the RepB nick site) fluorescently labeled with Cy5 at its 3′ end was mixed with 7.5 pmol of the protein. The reaction was performed in 20 µL of reaction buffer 8 (20 mM Tris-HCl pH 8.0) supplemented with 300 mM NaCl and 2 mM MnCl_2_. The mixture was incubated for 15 s or 5 min at 37°C, and SDS was subsequently added to a final concentration of 0.2%. Prior to electrophoresis, the samples were mixed with the appropriate volume of 10× DNA loading buffer (60% glycerol and 10 mM EDTA) and heated at 95°C for 3 min. The products were separated on 10% PAA (19:1 acrylamide:bis-acrylamide), 0.3% SDS denaturing gels. After electrophoresis, the gels were analyzed by using a FLA-3000 (FUJIFILM) imaging system (λ_ex_ 633 nm; λ_em_ 675 nm), and the fluorescence intensity of each band was quantified with the Quantity One software (Bio-Rad). After visualization of the fluorescent reaction products, the same gel was used for western blotting analysis with RepB antiserum. For that purpose, a Trans-Blot^®^ Turbo™ system was employed and a conventional semi-dry blotting protocol was followed for western blotting. Prior to transfer, the gel and the PVDF membrane were equilibrated in Towbin buffer (25 mM Tris, 192 mM glycine pH 8.3, 20% methanol) for 10 min. Once the blotting sandwich was assembled, the transfer was carried out by applying 25 V constant voltage for 30 min. Then, the membrane was washed with Tris-buffered saline (50 mM Tris-HCl pH 7.5, 150 mM NaCl) containing 0.1% Tween-20 (TTBS) and incubated with the appropriate dilution of the primary RepB antiserum in TTBS supplemented with 0.2% casein. After at least 2 h of incubation with gentle agitation, the membrane was washed with TTBS and further incubated for 2 h with the appropriate dilution of the peroxidase-conjugated anti-rabbit IgG secondary antibody. Finally, the membrane was washed with TTBS to remove the remaining secondary antibody, and incubated with an ECL western susbtrate for chemiluminescence detection. The membrane was visualized with the aid of a chemiluminescent detector Fuji LAS-3000.

### Analysis of the Nicking and Strand-Transfer Activities on Single-Stranded Oligonucleotides.

For the cleavage assays, 5 pmol of the 27-mer oligo substrate fluorescently labeled with Cy5 at its 3′ end was mixed with 150 pmol of an unlabeled 30-mer oligo (5′-TAC​TGC​GGA​ATT​CTG​CTT​CCA​TCT​ACT​ACG-3′) that provides the 3′-OH substrate for strand transfer. The mixture was incubated for 1 min at 25°C with OBD (0.1 pmol) in 20 μL of the appropriate reaction buffer. For reactions at pH 8.0 and 7.0, reaction buffers 8 and 7 (20 mM Tris-HCl adjusted to pH 8.0 and 7.0, respectively) supplemented with 300 mM NaCl, 2 mM MnCl_2_ and 0.2 mg/ml BSA, were used. For reactions at pH 6.5, 6.0, 5.5, 5.0, and 4.5, reaction buffers 6.5, 6, 5.5, 5, and 4.5 contained 20 mM MES adjusted to the indicated pH value and supplemented with 300 mM NaCl, 2 mM MnCl_2_ and 0.2 mg/ml BSA. Protein samples were previously diluted in 20 mM Tris-HCl buffer (pH 8.0) supplemented with 430 mM NaCl and 0.2 mg/ml BSA. The presence in the reaction mixtures of a 30-fold molar excess of the 30-mer oligo relative to the 27-mer oligo makes rejoining of the cleaved 27-mer oligo highly unlikely. After incubation for 1 min at 25°C, the reaction mixtures were treated with proteinase K (0.1 mg/ml) and 0.2% SDS for 15 min at 37°C.

To evaluate the strand-transfer activity of OBD at different pHs, the protein and the 27-mer oligo substrate were incubated at a 1:1 molar ratio in reaction buffer 7 at 25°C for 1 min. Then, the reaction mixture was diluted 10 times in buffers 7, 5, and 4.5, and an aliquot of this dilution was taken as a reference. Next, the unlabeled 30-mer oligo was added at a 50-fold molar excess relative to the initial 27-mer oligo and the strand-transfer reaction proceeded for 5 min at 25°C. After incubation, an aliquot of the three reactions mixtures was taken and, together with the reference aliquot, treated with SDS and proteinase K as stated above. Prior to electrophoresis, the samples were mixed with an appropriate volume of 10× DNA loading buffer and denatured by heating at 95°C for 3 min. The products were separated on 20% PAA (19:1 acrylamide:bis-acrylamide), 8 M urea denaturing gels. After electrophoresis, the fluorescent reaction products were visualized as depicted in Identification of the RepB-DNA Covalent Adduct.

### Analysis of the Effect of Temperature, Depletion of Divalent Cations and pH on the Stability of the OBD-DNA Covalent Adduct.

The OBD-DNA adduct was formed by mixing 5 pmol of the fluorescently labeled 27-mer oligo substrate with 7.5 pmol of OBD in 10 µL of buffer 7. The mixture was incubated for 2 min at 25°C and subsequently diluted 30 times with the same reaction buffer equilibrated to the reaction temperature. To analyze the effect of the temperature, diluted reactions were incubated at 0°C, 22°C or 37°C for up to 1 h, and aliquots (10 µL) were taken at intervals. The effect of the depletion of Mn^2+^ ions was analyzed by treating the diluted reaction with 20 mM EDTA and incubating the mixture at 22°C, with 10-µL aliquots being withdrawn at intervals over a 1-h period. The stability of the covalent adduct at different pHs was analyzed by a similar procedure. The covalent complex was formed in buffer 7 as described above. After being treated with 10 mM EDTA for 1 min at 25°C, the reaction mixture was diluted 30 times in the corresponding reaction buffer adjusted to the pH of analysis (buffers 7, 6, 5.5, 5 or 4.5) and incubated 5 min at 4°C. Next, the mixture was incubated at 25°C and 10-µL aliquots were withdrawn at intervals over a 1-h period. In all cases, an initial *t = 0* aliquot was also taken. All aliquots were treated with proteinase K (0.1 mg/ml) and 0.2% SDS for 15 min at 37°C. Samples were analyzed by electrophoresis and the gels visualized as depicted in Identification of the RepB-DNA Covalent Adduct.

The half-life time (*t*_*1/2*_) of the covalent complex, defined as the time it takes for a 50% reduction of the adduct fraction, was estimated according to the equation:

[adductt]/[adductt0]=1/2tt1/2 (1)

where $\left[adduct_{t}\right]$ and $\left[adduct_{t_{0}}\right]$ represent the fraction of the nicking products in the form of adduct at time *t* and *t* = *0*, respectively.

This equation can be converted into a linear function by taking logarithms:

$$\log\left(\left[adduct_{t}\right]/\left[adduct_{t_{0}}\right]\right)=\;-tlog2/t_{1/2}$$ (2)

The log([*adduct*_t_]/[adduct_t0_]) was plotted against time (*t*), and the experimental data were fitted to Eq. 2 by linear regression. The term −*log2/t*_*1/2*_ represents the slope (*m*) of the fit, so that the half-life time (*t*_*1/2*_) can be calculated from the expression:

$$t_{1/2}=\;-log2/m\;$$ (3)

### Preparation of Total Protein Extracts and Detection of RepB by Western Blotting

*L. lactis* MG1363*/*pMV158 was grown for 10 and 20 generations in Tc-free M17′ adjusted to pH 7.0, 5.0 or 4.5, and aliquots (4 ml) of the cultures were taken when they reached an OD_660_ of 0.4. Cells were harvested by centrifugation and resuspended in 300 µL of lysis solution (50 mM Tris-HCl pH 7.0, 0.3% SDS, 200 mM DTT, 3 mM MgCl_2_, 16 μg/ml of protease-free RNase I and 60 μg/ml of protease-free DNase I). Cell suspension was transferred to 2-mL tubes containing 0.1 mm silica spheres lysing matrix (FastPrep^®^) and the cells were lysed by beating the tubes at 6 m/s for 45 s at 25°C with the aid of a Hybaid Ribolyser homogenizer. The cell debris was removed by centrifugation at 14,000 × g for 20 min at 4°C. Total protein concentration of the extracts was determined with a Qubit fluorometer by using the Qubit Protein dsDNA Assay kit (Molecular Probes). Similar total protein concentrations (maximum difference of 11%) were obtained across the extracts. Samples were analyzed by electrophoresis on 12% SDS-glycine-PAA gels and transferred to a PVDF membrane for western blotting analysis with RepB antiserum as described in Identification of the RepB-DNA Covalent Adduct. Images of the membrane were analyzed with the Quantity One (Bio-Rad) software and the luminescent bands corresponding to the RepB protein were quantified.

The total protein amount loaded for western blotting was controlled in two different ways: 1) by adjusting the volume of protein extracts loaded in the same gel in order to keep constant the amount of total protein in the different samples analyzed; and 2) by normalization to an isolated non-specific band (internal control) that corresponds to a chromosome-encoded protein whose concentration remained rather constant across the different extracts. Similar results were obtained when comparing the RepB levels between protein extracts from cells grown at different pHs, irrespective of whether normalization to the internal control band was made or not.

### Statistical Analysis

Statistical analyses were carried out using SigmaPlot software (v12.5, Systat software, San Jose, CA, United States). One-way ANOVA tests were conducted to analyze the effect of pH on the OBD endonuclease activity, the PCN and the bacterial growth rate, as well as the effect of pH and temperature on the adduct half-life time. Student t-tests were used to compare the relative RepB level and the RepB to pMV158 ratio in *L. lactis* MG1363/pMV158 cells grown at different conditions with respect to those of the reference condition (10 generations at pH 7.0). The level of significance in both statistical analyses was set at *p* < 0.05.

## Results

### RepB-mediated DNA cleavage at the nick site renders a transient covalent nucleoprotein complex.

Direct verification of the existence of a RepB-DNA covalent intermediate in DNA cleavage and rejoining has largely remained elusive and only indirect indications could be reported for a long time (Moscoso et al., 1995; Moscoso et al., 1997). Recently, we succeeded in detecting a product arising from the RepB endonuclease activity at the nick site that corresponded to the 3′-fragment of a 3′-end fluorescently labeled substrate oligonucleotide and exhibited a decreased electrophoretic mobility relative to the corresponding protein-free DNA product on 20% PAA-urea gels. This slowly migrating product could only be observed when the temperature and time of incubation of the substrate DNA with either RepB_6_ or OBD were reduced with respect to previously-reported conditions (from 60°C to ≤37°C and from 30 min to ≤15 min, respectively), and after treatment of the reaction products with 0.2% SDS and 0.1 mg/ml proteinase K (Ruiz-Masó et al., 2016). The anomalous migration of the labeled reaction product (which actually appears as a set of 2–3 bands) was hypothesized to arise from a small protein polypeptide that remained bound to the 3′-fragment generated upon cleavage of the substrate oligonucleotide by the RepB_6_ or OBD protein. The discrete bands assigned to this putative covalent complex were shown to result, at least in part, from partial digestion of the initiator protein with proteinase K (not shown). Moreover, we observed that once the substrate utilization had gone to completion, further incubation of the reaction mixture resulted in the decay of the slowly migrating DNA product and the concomitant rise of its protein-free counterpart (see below). All these observations suggested that, upon RepB-mediated specific cleavage at the nick site, a covalent link between the protein and the 5′-phosphate end of the cleaved DNA was formed that dissociated rapidly.

**FIGURE 1:**
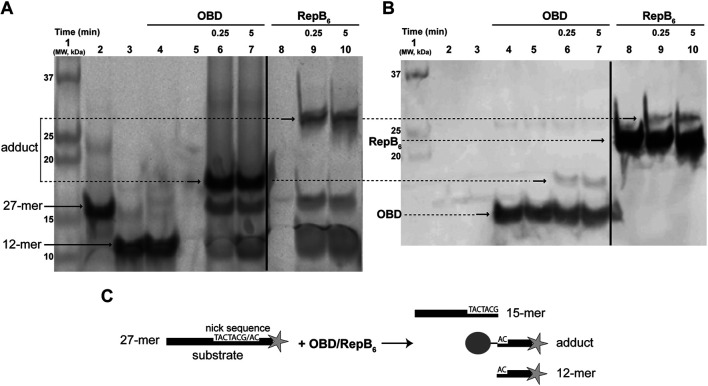
Detection of the RepB-DNA covalent complex. **(A)** Direct visualization of the fluorescent DNA species that arise from the endonuclease activity of RepB_6_ and OBD on the ssDNA 27-mer oligo substrate. A prestained and chemiluminescent protein ladder was loaded in lane 1. Lanes 2 and 3 show the migration of fluorescent 27-mer substrate and chemically-synthesized 12-mer oligo, respectively. In lane 4, a mixture of OBD and chemically-synthesized fluorescent 12-mer oligo (which is not a substrate of the protein) was loaded. OBD (lanes 6 and 7) and RepB_6_ (lanes 9 and 10) were incubated for the indicated times with the 27-mer substrate. The slower migrating bands corresponding to covalent complexes between RepB_6_ or OBD and the fluorescent 12-mer DNA generated by cleavage of the 27-mer substrate are indicated. The dividing line indicates grouping of different parts of the same gel. **(B)** Detection of RepB_6_ and OBD in the gel shown in panel A by chemiluminescent western blotting employing anti-RepB polyclonal serum. Lanes 5 and 8 contain OBD and RepB_6_ proteins, respectively. The position of the slower migrating chemiluminescent bands that correspond to covalent complexes between RepB_6_ or OBD and the 12-mer oligo is indicated. The presence of DNA-free protein subunits was also detected and their position indicated. The dividing line indicates grouping of images of the same gel as indicated in panel **(A)**. **(C)** Schematic description of the endonuclease reaction mediated by RepB_6_ or OBD.

To ascertain the nature of the transient nucleoprotein complex resulting from the RepB-mediated cleavage of the 27-mer oligo substrate (which contains the nick sequence and is fluorescently labeled at its 3′-end), the reaction products were subjected to two different analyses. The reactions were performed by incubating the 27-mer oligo DNA with a 1.5-fold molar excess of either RepB_6_ (consisting of six 24.12 kDa polypeptide chains) or OBD (a single 16.4 kDa polypeptide chain) at 37°C for the indicated times. After stopping the reactions with 0.2% SDS, the mixtures were processed as described (Identification of the RepB-DNA Covalent Adduct in Materials and Methods). Treatment with SDS denatures proteins, dissociates the oligomeric structures and confers uniform negative charge densities to the polypeptides, thus allowing the electrophoretic separation of covalent nucleoprotein complexes according to their molecular sizes. The fluorescent DNA species were visualized with the Phosphorimager FLA-3000 system (Figure 1A). In addition to the 27-mer oligo substrate and to the 12-mer oligo (the protein-free fluorescently-labeled final product of the RepB_6_/OBD-mediated cleavage), slower-migrating fluorescent DNA species were observed whose electrophoretic mobilities were inversely related to the polypeptide chain length of the protein employed (RepB_6_ or OBD; Figure 1A). We speculated that these retarded bands could correspond to covalent complexes between the target RepB or OBD protein and the fluorescent 12-mer DNA generated by cleavage of the 27-mer substrate oligonucleotide. To confirm this hypothesis, once the fluorescent bands corresponding to DNA and nucleoprotein complexes were imaged (Figure 1A), free RepB/OBD proteins and RepB/OBD-DNA complexes in the gel were detected by chemiluminescent western blotting employing anti-RepB polyclonal serum as the primary antibody and peroxidase-conjugated anti-rabbit IgG as secondary antibody (Figure 1B). Chemiluminescent signals appeared linked to the fluorescent DNA species that exhibited retarded mobility, which were thus confirmed to correspond to covalent (SDS-resistant) complexes between the 12-mer oligo and either RepB or OBD (see reaction scheme in Figure 1C). Apart from these nucleoprotein complexes, chemiluminescent bands that also displayed electrophoretic mobilities inversely related to the length of the target protein polypeptide chain were observed in samples containing RepB_6_ or OBD, irrespective of the presence or absence of DNA (either the 27-mer substrate or the 12-mer product oligo), and were ascribed to the DNA-free polypeptides of the corresponding protein (Figure 1B).

Given that the OBD domain retains the specific endonuclease and strand-transfer activities of the RepB protein, we chose to employ OBD, which is produced more easily due to the presence of the His-tag, in all subsequent experiments throughout this work.

### Low pH Impairs the Endonuclease Activity of OBD

**FIGURE 2:**
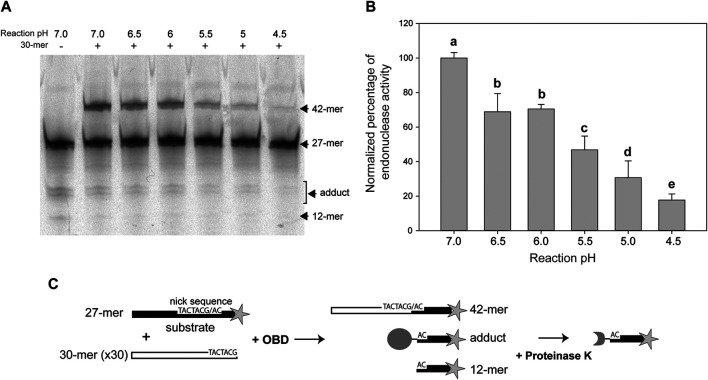
Endonuclease activity of OBD at different pHs. **(A)** Reaction product pattern generated by the nicking and strand-transfer activities of OBD on ssDNA oligos at the indicated reaction pHs. The 27-mer oligo substrate (500 nM), labeled at its 3′-end with the fluorescent dye Cy5 (indicated by a star in panel **(C)**), and a 30-fold molar excess of the unlabeled 30-mer oligo were incubated with the protein (10 nM) at 25°C for 2 min. The resultant fluorescent oligos were analyzed by electrophoresis in 20% PAA-urea gels, and visualized with the aid of a FLA 3000 (FUJIFILM) imaging system. A representative gel from one of four independent experiments is displayed. The bands corresponding to the 12-mer DNA generated by cleavage of the 27-mer substrate and to the 42-mer DNA strand-transfer product are indicated. The covalent adduct is represented by a set of 2–3 bands that arise from partial digestion of OBD with proteinase K. **(B)** Vertical bar graph showing the percentage of reaction products rendered by OBD at the indicated pHs. The sum of all reaction products was considered for catalytic activity quantification. The percentage of activity obtained at pH 7.0 was used as a reference for normalization. Vertical bars represent the average value of four independent experiments. Error bars represent standard deviations. Average values with different lowercase letters were significantly (*p* < 0.05) different. **(C)** Schematic description of the different reaction products.

The DNA cleavage activity of OBD was assayed in the presence of a 50:1 molar ratio of the 27-mer oligo substrate to the enzyme. To avoid regeneration of the substrate oligo by the strand-transfer activity of the protein, which would entail an underestimation of its cleavage activity, a 30-fold molar excess of the unlabeled 30-mer oligo (carrying the nick sequence 5′ to the nick site) relative to the fluorescently-labeled 27-mer oligo was added to the reaction mixture. In this way, virtually the only strand-transfer product should correspond to the 42-mer oligo that arises from joining of the 30-mer oligo with the 3′-fluorescently labeled 12-mer cleavage product and is thus distinguishable from the substrate 27-mer oligo (Figures 2A,C).

In previous works, all assays of the catalytic activity of the RepB protein or its OBD domain had been carried out at a pH value of 8.0. This pH was chosen based on early reported conditions used for analyzing the activity of the plasmid pT181 Rep protein (Koepsel et al., 1985), which, like RepB, catalyzes DNA cleavage and rejoining reactions mediated by nucleophilic attacks. In the present work, we aimed to analyze the pMV158 replication capacity and the RepB catalytic activities in a range of pHs, varying from neutral (7.0) to acidic (4.5) values, at which LAB usually grow (Schillinger et al., 2006). Thus, we first compared the endonuclease activity of OBD at pHs 8.0 and 7.0, and did not found significant differences between both conditions (not shown). All subsequent *in vivo* and *in vitro* experiments were performed at pH values of 7.0, 6.5, 6.0, 5.5, 5.0, and 4.5, and the data obtained were normalized with respect to those obtained at pH 7.0, to which 100% activity was assigned.

The endonuclease activity of OBD clearly decreased with decreasing pH values, the activity at pH 4.5 becoming ∼16% of that observed at pH 7.0. Statistically significant decreases were found between all pH values, except for pHs 6.5 and 6.0 (Figure 2B).

### The Strand-Transfer Activity of OBD is Not Significantly Affected Over a Wide pH Range.

**FIGURE 3:**
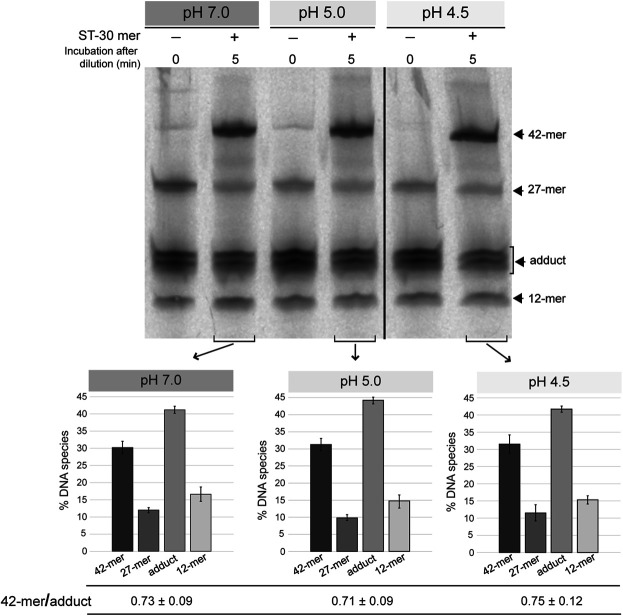
Strand-transfer activity of OBD at different pHs. Gel showing the reaction products that arise from the strand-transfer activity of OBD at different pHs. After setting up a nicking reaction (OBD:27-mer molar ratio of 1:1) in reaction buffer 7 at 25°C for 1 min, the mixture was diluted 10 times in buffers 7, 5.0, and 4.5. Next, the unlabeled 30-mer oligo was added to a 50-fold molar excess relative to the initial 27-mer oligo and the strand-transfer reaction was allowed to proceed for 5 min under the same conditions used for the nicking reaction. Aliquots of the three reactions were taken and analyzed. Aliquots of the diluted reactions before adding the 30-mer oligo were also analyzed and used as reference. The bands corresponding to the 12-mer DNA generated by cleavage of the 27-mer substrate and the 42-mer strand-transfer product are indicated (see reaction scheme in Figure 2C). The covalent adduct is represented by a set of 2–3 bands that arise from partial digestion of OBD with proteinase K. A representative gel of three different experiments is displayed. Below the gel image, three bar graphs represent the quantification of the different DNA species generated at the three reaction pHs analyzed. Vertical bars represent the average value of three different experiments and error bars represent standard deviations. The ratio (mean ± SD) between the percentages of the 42-mer product and the adduct for each quantification is also shown.

To test the strand-transfer activity of the RepB OBD domain, the protein and the 3′-fluorescently labeled 27-mer oligo substrate were incubated at a 1:1 molar ratio, in reaction buffer 7, at 25°C, until reaction products (mainly consisting of the covalent adduct) reached 80–90% of total DNA. After diluting 10 times the reaction mixture in buffers at each of the pHs studied (7.0, 5.0, and 4.5), the unlabeled 30-mer oligo was added at a 50-fold molar excess relative to the initial fluorescently-labeled 27-mer substrate and the strand-transfer reaction was allowed to proceed for 5 min at 25°C. Similar ratios of the 42-mer product to the OBD-DNA adduct were obtained irrespective of the pH, showing that the strand-transfer activity of the protein is not significantly affected by the acidic conditions of the reaction mixture (Figure 3).

Preservation of the strand-transfer activity of the RepB OBD domain even at the most acidic conditions analyzed suggests that the active center of the protein shows structural stability in the pH range from 8.0 to 4.5.

### Low pH Improves the Stability of the OBD-DNA Covalent Adduct.

**FIGURE 4:**
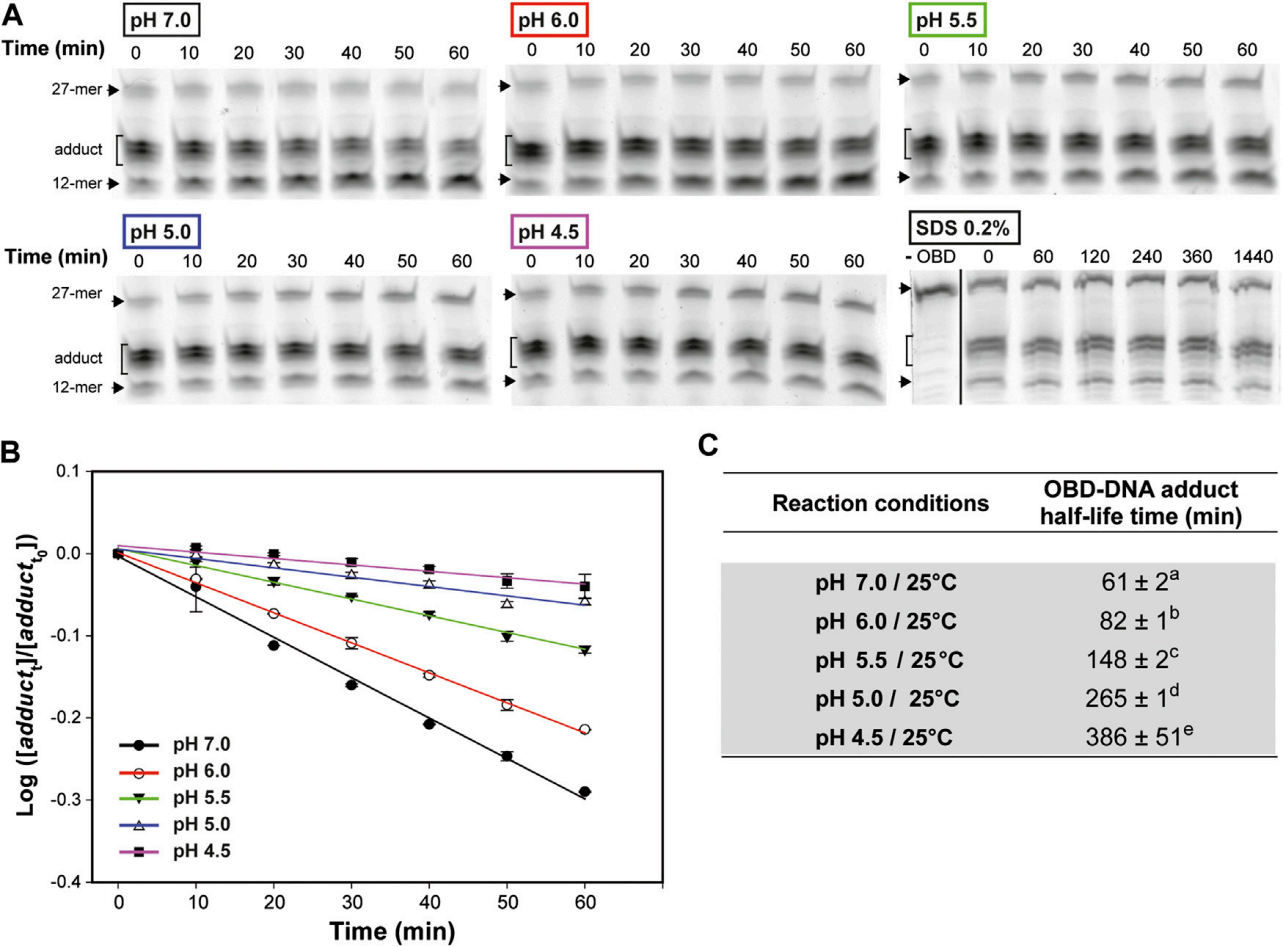
OBD-DNA adduct stability at different pHs. **(A)** Gels showing the effect of pH on the time course of the adduct-hydrolysis reaction. The reaction mixtures contained a 1.5:1 molar ratio of OBD to the 3′-fluorescently labeled 27-mer oligo substrate and were prepared in buffer 7. After 2 min of incubation at 25°C, the endonuclease reaction was stopped with 10 mM EDTA before diluting the mixtures in buffer 7, 6, 5.5, 5 or 4.5 for 1-h incubation at the same temperature. Several aliquots were taken every 10 min and analyzed in 20% PAA-urea gels. A gel showing the effect of denaturing the protein on the stability of the adduct is also shown. In this case, the endonuclease reaction was performed in buffer 7 for 2 min at 22°C, and then the mixture was diluted in buffer 7, treated with 0.2% SDS and incubated at the same temperature for 24 h. Several aliquots were taken at the indicated times and analyzed in 20% PAA-urea gels. A control lane (-OBD) showing the migration of the fluorescent 27-mer substrate is included. The dividing line indicates grouping of different parts of the same gel. **(B)** Kinetics of the hydrolysis of the OBD-DNA adduct at different pHs. The logarithm of the ratio between the fraction of adduct at each of the time intervals analyzed (*adduct*_t_) and the initial fraction (*adduct*_t0_) was plotted against time, and the experimental data were fitted to Eq. 2 by linear regression. The OBD-DNA adduct half-life time was calculated from the slope of the regression curve according to Eq. 3. Plots display the adduct decay curves obtained from at least five independent experiments for each pH, with the symbols and vertical bars representing the mean and standard deviation, respectively. **(C)** Table showing the adduct half-life time calculated from at least five independent experiments performed under the indicated reaction conditions. The values are expressed as mean ± SD. Values with different superscript letters were significantly (*p* < 0.05) different.

To accurately determine the influence of the pH in the stability of the covalent link between OBD and the 5′-phosphoryl group generated upon cleavage of the oligo substrate, we first tested the optimal temperature conditions for these experiments. To this end, reaction mixtures that contained a 1.5:1 molar ratio of OBD to the 3′-fluorescently labeled 27-mer oligo were prepared in buffer 7 and incubated at 25°C for 2 min, so that the oligo substrate was almost completely (>80%) exhausted. Then, the mixtures were diluted 30 times with the same buffer 7 and further incubated at either 0°C, 22°C or 37°C to analyze the change in the adduct fraction over time. A sample of the diluted mixture was brought to 20 mM EDTA and incubated at 22°C in order to study whether depletion of Mn^2+^ affected the stability of the OBD-DNA covalent linkage. Moreover, the effect of denaturing the protein on the stability of the adduct was analyzed by adding 0.2% SDS to a sample of the freshly-diluted mixture and incubating at 22°C for 24 h. The half-life time of the adduct, which measures its stability, was calculated according to Eq. 3. Hydrolysis of the adduct was found to be prevented in the presence of SDS (Figure 4A), indicating that this process is catalyzed by the protein and requires the native folding of the OBD. The stability of the covalent adduct clearly decreased with increasing temperatures, the half-life at 0°C (∼220 min) being about 2.5- and 10-fold longer than those determined at 22°C (∼84 min) and 37°C (∼25 min), respectively (Supplementary Figure S1). On the other hand, chelation of Mn^2+^ did not affected significantly the stability of the adduct (Supplementary Figure S1). We finally chose to investigate the effect of the pH on the stability of the OBD-DNA adduct at a temperature of 25°C, which can be easily controlled in a heating block and was expected to confer a medium stability to the covalent nucleoprotein complex.

To test the effect of the pH on the stability of the OBD-DNA adduct, reaction mixtures containing a 1.5:1 molar ratio of OBD to the 3′-fluorescently labeled 27-mer oligo were prepared in buffer 7, incubated at 25°C for 2 min and treated with 10 mM EDTA before being diluted 30 times in buffer 7, 6, 5.5, 5 or 4.5 for further incubation at the same temperature (Figures 4A,B). A remarkable and statistically significant stabilization of the OBD-DNA adduct was observed as the pH was decreased, with the half-life of the covalent complex increasing from ∼1 h (at pH 7.0) to more than 6 h (at pH 4.5) (Figure 4). Hence, it seems that acidity greatly impairs hydrolysis of the adduct catalyzed by the protein.

### The PCN is Inversely Related to the Growth Rate of *L. lactis* MG1363/pMV158 Cells That Grow in Tc-Containing Media Adjusted to Different pHs.

Plasmid pMV158 has been previously shown to be stably inherited and not to cause any statistically significant decrease in the growth rate of *L. lactis* MG1363 host cells growing in M17 medium supplemented with 4% glucose. Conversely, when the lactococcal host cells were grown in M17 supplemented with 4% glucose and 1 μg/ml Tc (the resistance to which is encoded by pMV158), the bacterial growth rate underwent a statistically significant 25% reduction (Garay-Novillo et al., 2019). Since expression of the plasmid-encoded *tetL* gene has been reported to be constitutive (Lacks et al., 1986), the decrease in the growth rate of the bacterial host was attributed directly to the presence of the antibiotic, which was thought to cause some noxious effect before being expelled from the cell. In the lactococcal host cells grown under these latter conditions (i.e., in M17 plus glucose and Tc), pMV158 exhibited ∼18 copies per chromosome equivalent (Garay-Novillo et al., 2019). The copy number of pMV158 may vary, however, between host cells growing in different media, as it has been reported that the PCN shows a strong dependence on the bacterial growth rate and that, in general, the plasmid concentration decreases with increasing growth rates (Summers, 1996; Klumpp, 2011; Ruiz-Masó et al., 2017). For this reason, we first determined the PCN and the bacterial growth rate in *L. lactis* MG1363/pMV158 cells cultured in M17′ adjusted to different pHs and supplemented with 1 μg/ml Tc.

The growth rate of the lactococcal host decreased abruptly as the acidity of the culture medium was increased, and approached to 0 at pH 4.5 (*μ* = 0.05 ± 0.02; Table 1). This growth slowdown at the lowest pHs was much more pronounced than that previously reported for the plasmid-free *L. lactis* MG1363 cells, whose growth rate at pH 4.5 was only decreased by ∼50% with respect to pH 6.5 (Mercade et al., 2000). Since pMV158 does not seem to burden significantly the lactococcal host by itself (Ruiz-Masó et al., 2017), we speculate that the strong pH-dependence of the growth rate of the *L. lactis* MG1363/pMV158 cells growing in the presence of Tc arises from the antibiotic efflux mechanism mediated by the plasmid-encoded TetL protein, which exchanges Tc-divalent metal complexes for protons (Krulwich et al., 2001). Hence, the activity of the pMV158-encoded Tc^R^ determinant could further increase the intracellular acidity of the lactococcal host grown at low pHs, causing acid stress damage of the DNA (Jeong et al., 2008). This hypothesis is supported by the observations that: 1) a significant fraction of the plasmidic DNA in the lactococcal cells grown at pH 5.0 and especially at pH 4.5 in the presence of Tc, but not in the absence of the antibiotic, is found as open circular forms; and 2) a smear clearly appears in the gDNA extracted from the lactococcal cells grown at pH 4.5 in the presence, but not in the absence, of Tc (not shown).

**TABLE 1:** pMV158 PCN and growth rate of *L. lactis*/pMV158 cells grown in Tc-containing M17’ adjusted to different pH values.

M17′ pH	µ (h^−1^) M17′ + Tc (mean ± SD)	pMV158 copy number (mean ± SD)	
		Gel quantification	qPCR quantification
7.0	0.58 ± 0.06^α^	7.2^A^	7.2 ± 2.0^a^
6.5	0.34 ± 0.02^β^	9.6 ± 0.8^A^	9.5 ± 1.0^a^
6.0	0.30 ± 0.03^β,γ^	10.5 ± 1.7^A^	11.8 ± 1.2^a^
5.5	0.24 ± 0.03^γ^	23.4 ± 3.0^B^	23.5 ± 0.8^b^
5.0	0.15 ± 0.03^δ^	41.3 ± 4.4^C^	43.1 ± 2.3^c^
4.5	0.05 ± 0.02^θ^	47.9 ± 4.2^C^	52.9 ± 4.3^d^

The number of pMV158 plasmid molecules per chromosome equivalent was determined by qPCR or by densitometric analysis of agarose gels. In this latter method, the plasmidic to chromosomal DNA ratios were compared with that obtained in the lactococcal host cells grown at pH 7.0 in the presence of Tc, to which the corresponding qPCR-determined pMV158 PCN value (red color number) was assigned. The copy number and growth rate values given in the table are expressed as mean ± standard deviation of three independent experiments (biological samples). All the values with the same superscript letter were not found to have statistically significant differences.

The pMV158 PCN per chromosome equivalent in *L. lactis* MG1363/pMV158 cells grown at different pHs in the presence of Tc was inversely correlated with the bacterial growth rate. In fact, the PCN in cells of the lactococcal host grown in Tc-containing M17′ at pH 4.5 was ∼7.5-fold higher than that in the host cells grown at pH 7.0 (Table 1). Increase in the PCN as the bacterial growth slows down has been reported for theta-replicating plasmid R1, which also encodes a Rep initiator protein (Engberg and Nordström, 1975). Also, a 3.5-fold increase in the PCN was observed for RCR lactococcal plasmid pLd3 at near-zero growth rates of the host cells (van Mastrigt et al., 2018).

### Plasmid pMV158 is Stably Inherited by the Lactococcal Host Cells Irrespective of the pH of the Culture Medium.

**FIGURE 5:**
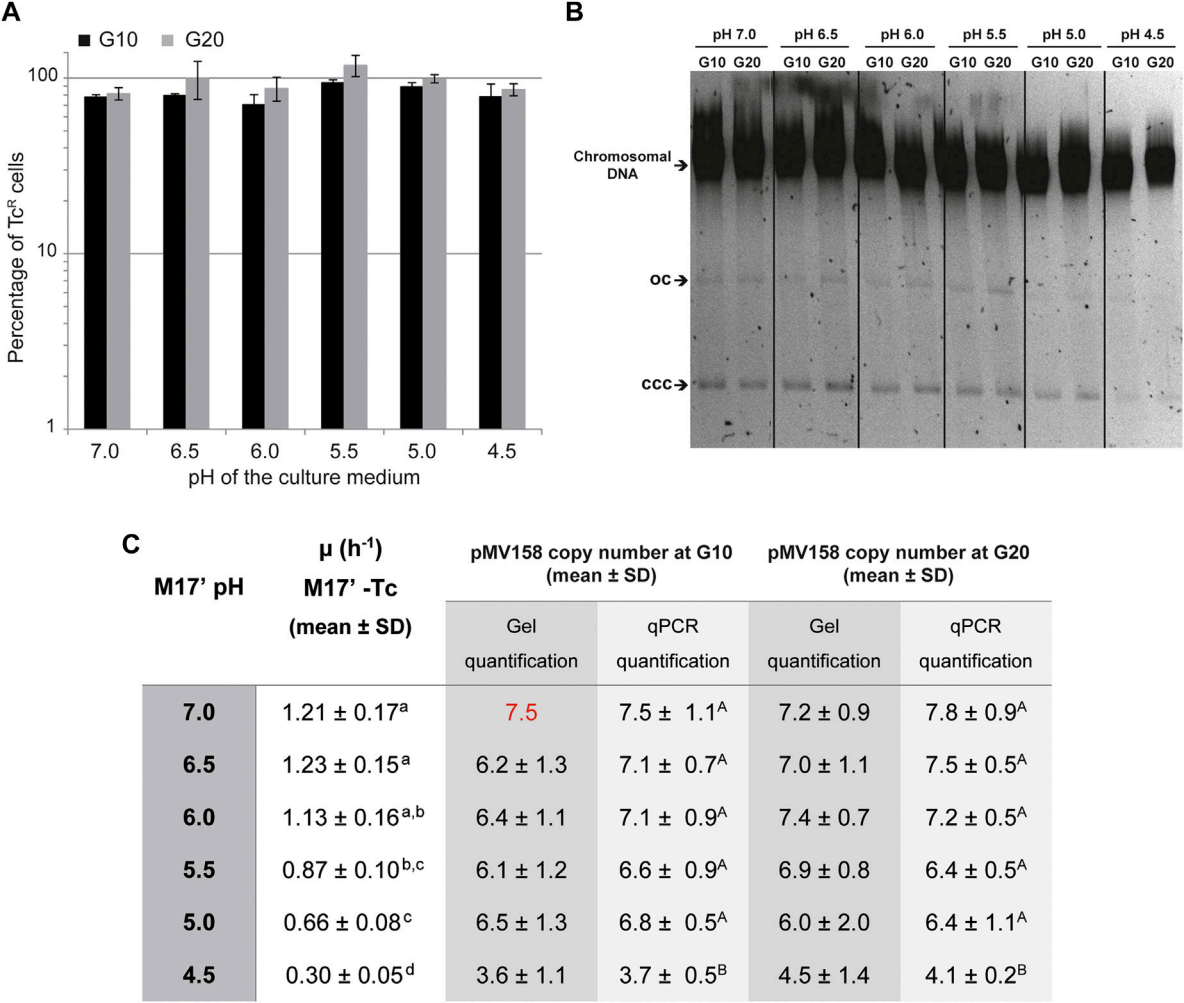
Segregational stability and PCN of pMV158 in *L. lactis* grown in M17′ adjusted to different pHs. **(A)** Analysis of the segregational stability of plasmid pMV158 in *L. lactis* MG1363. The stability of inheritance of pMV158 was analyzed after growing the lactoccocal cells for 10 and 20 generations in M17′ adjusted to different pH values in the absence of selective pressure. The vertical bars represent an estimation of the percentage of Tc resistant (Tc^R^) cells after 10 (black bars) and 20 (gray bars) generations at the indicated pHs. Bar height represents the average value of three different analysis and error bars are standard deviations. **(B)** The gel shows the total DNA content of the *L. lactis* MG1363/pMV158 strain grown at the indicated pHs for 10 and 20 generations in the absence of Tc. Supercoiled monomeric (CCC) and open circle (OC) forms of pMV158 are indicated in the gel. Dividing lines indicate grouping of different parts of the same gel. This image is representative of at least three agarose gels used in the analysis and quantification of the DNA bands for PCN determination. **(C)** The table shows the pMV158 PCN in the lactococcal cells grown for 10 and 20 generations in Tc-free M17′ adjusted to different pHs. The pMV158 PCN per chromosome equivalent was determined by qPCR or by densitometric analysis of stained agarose gels. In this latter method, the plasmidic to chromosomal DNA ratios were compared with the one obtained in the lactococcal host cells grown at pH 7.0 for 10 generations, to which the corresponding qPCR-determined PCN value (red color number) was assigned. The table also includes the growth rates of *L. lactis*/pMV158 in Tc-free M17′ media adjusted to the different pH values. The copy number and growth rate values given in the table are expressed as mean ± SD of three independent experiments (biological samples). All the values with the same superscript letter were not found to have statistically significant differences. The statistical analysis of the PCN was only performed for the results obtained by qPCR, for which a lower standard deviation was found.

A first evaluation of the capacity of pMV158 to replicate in the lactococcal host cells growing in media with different pHs was carried out by analyzing its segregational stability over a period of 10 and 20 generations in the absence of selection for the plasmid-encoded antibiotic resistance marker (Tc^R^). The plasmid was maintained stably, irrespective of the pH of the culture medium, indicating that regulated replication was feasible in all cases (Figures 5A,B).

### The Copy Number of pMV158 Decreases in Lactococcal Cells Grown at the Lowest pH.

A second approach to evaluate the capacity of pMV158 to replicate in lactococcal host cells that grow at different pHs consisted in determining the copy number of the plasmid after 10 and 20 generations in the absence of Tc. The growth rate of *L. lactis* MG1363/pMV158 in the different media was also determined to find out whether the foreseeable slowdown at the lowest pHs resulted in an increase of the PCN, as was the case with the host cells that grew in the presence of Tc (see The PCN is Inversely Related to the Growth Rate of *L. lactis* MG1363 /pMV158 Cells That Grow in Tc-Containing Media Adjusted to Different pHs).

Compared with the medium containing Tc, the growth of *L. lactis* MG1363/pMV158 in antibiotic-free medium showed a weaker dependence on the pH, with growth rates ranging from ∼1.1-1.2 (at pHs 7.0, 6.5, and 6.0) to 0.3 (at pH 4.5) (Figure 5C).

The pMV158 copy number per chromosome equivalent showed only a rather small but statistically significant decrease (by ∼50%) at the lowest pH (Figure 5C). The PCN reduction in the lactococcal cells grown in acidic media was observed irrespective of whether the gDNA was analyzed by qPCR or by densitometric analysis of plasmidic and chromosomal DNA bands on stained agarose gels (Figure 5B).

We have observed that the overall yield of gDNA from cells grown at different pHs to the same OD is quite similar. Moreover, since the cell density is kept constant, the mass and volume of the bacterial cells have the same dependence on the OD of the culture (Klumpp, 2011). Hence, it can be inferred that lactococcal cells grown at different pH conditions have a similar intracellular concentration of the chromosome, which is used as a reference for the determination of the PCN. Taking this into account, we can conclude that the estimated PCN correlates directly with the concentration of plasmid molecules within the lactococcal host cells.

### The Molar Ratio of RepB to Plasmid DNA is Significantly Increased at a pH Value of 4.5.

**FIGURE 6:**
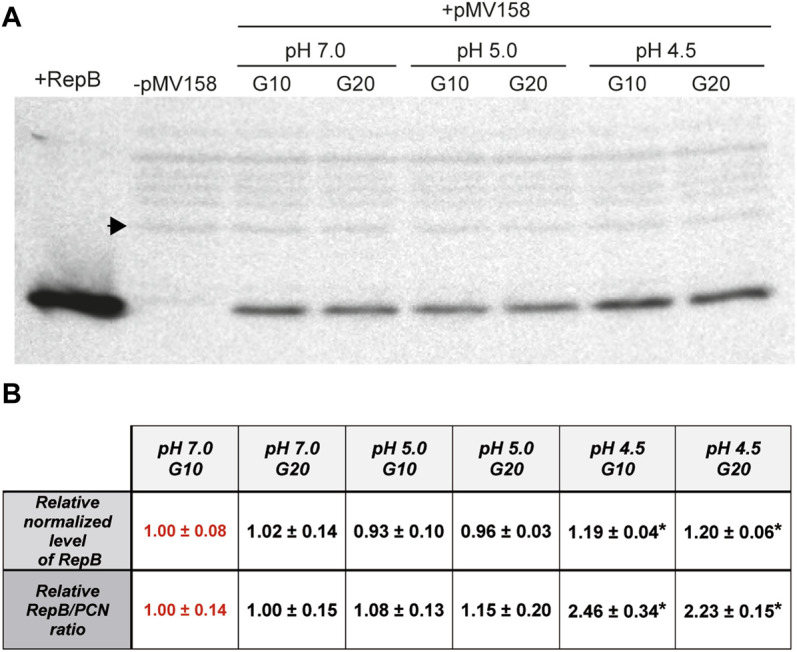
Estimation of the relative RepB concentration and RepB to pMV158 molar ratio in lactococcal cells grown at different pHs. **(A)** Western blot analysis with RepB antiserum of protein extracts from *L. lactis*/pMV158 cells grown for 10 and 20 generations in Tc-free M17′ adjusted to pH 7.0, 5.0, and 4.5. Pure RepB protein and a protein extract from a pMV158-free *L. lactis* culture (−) were used as controls. The sample volume of the protein extracts was adjusted in order to keep constant the amount of total protein loaded in the gel. A black arrowhead indicates the band used as an internal loading control. **(B)** The normalized RepB level (9.91 ± 0.77) and RepB/PCN ratio (1.32 ± 0.18) were taken as reference and assigned the value 1 (in red color) for comparison with the different conditions. Data are presented as mean ± SD of the analysis of four different western blot assays. Values with an asterisk were found to have statistically significant differences with the corresponding reference value.

The intracellular concentration of the initiator RepB protein in the lactococcal host grown in media adjusted to pH 5.0 and 4.5 relative to that at pH 7.0, which was taken as the standard condition, was analyzed by western blot of protein extracts obtained from bacterial cultures at each condition (Figure 6A). The intracellular level of RepB (normalized to the internal control) was virtually the same in cells grown for either 10 or 20 generations at pH 7.0, and that estimated for 10 generations was arbitrarily taken as reference and assigned the value 1 (Figure 6B). The RepB level in the host cells grown at pH 5.0 also did not differ significantly from that observed in the cells that grew at pH 7.0, whereas the relative protein concentration within the cells grown for 10 or 20 generations at pH 4.5 was ∼20% higher than the reference value and these differences were found to be statistically significant (Figure 6B). The RepB to pMV158 molar ratio relative to that within the host cells grown for 10 generations at pH 7.0 was next estimated for the lactococcal cells that had been grown for up to 20 generations at pH values of 7.0, 5.0, and 4.5. After 20 generations of growth at pH 7.0, the host cells presented the same RepB to plasmid molar ratio as the cells grown for 10 generations at neutral pH (reference value). Also, the protein to plasmid molar ratio in cells grown for 10 or 20 generations at pH 5.0 was close to that in cells that grew at pH 7.0. Remarkably however, the decrease of the pMV158 copy number in cells that grew for 10 or 20 generations at pH 4.5 occurred despite the increase in RepB concentration, so that the protein to plasmid molar ratio became ∼2.4-fold higher than the ratio estimated for the cells grown at the reference pH value (Figure 6B).

## Discussion

So far, there have been only a very few reports about the effect of the pH on the plasmid RCR, a process that is initiated and terminated by Rep-mediated nucleophilic reactions. In fact, there is a lack of information on the efficiency of the nucleophilic DNA cleavage catalyzed by Rep proteins of RCR plasmids, bacteriophages and eukaryotic viruses under acidic conditions. The susceptibility to low pH of the strand-transfer reaction, as well as the stability of the Rep-DNA adduct against hydrolysis, have not yet been tested either.

In the present work, we have undertaken a global analysis to determine whether the acidity affected both the different *in vitro* reactions catalyzed by the pMV158-encoded RepB initiator protein and the parameters (PCN and stability) that prove the *in vivo* replication ability of the plasmid. In this sense, the pMV158 system provides the advantage of its singular promiscuity, which enables the plasmid to replicate in a wide variety of hosts, many of them able to grow at quite low pH conditions (Schillinger et al., 2006; Ruiz-Masó et al., 2017). Importantly, these studies may help to optimize the use of pMV158-based vectors in LAB, which constitute a large and heterogeneous group of bacteria widely employed, among other things, for the manufacturing of fermented and/or functional foods, for the production of probiotics and for the design and development of mucosal vaccines (Juarez del Valle et al., 2014; Mohedano et al., 2014; Russo et al., 2014).

The endonuclease activity of RepB on the nick sequence, which takes place at both the replication initiation and termination steps, is severely impaired at low pHs (Figure 2). Conversely, the strand-transfer activity, which is essential for the generation of the circular ssDNA intermediate that is used as the template for lagging-strand synthesis, does not seem to be affected by the pH (Figure 3). Finally, hydrolysis of the RepB-DNA adduct, which is shown here to require preservation of the native structure of the protein and hence may be mediated by the catalytic center of RepB, decreases with decreasing pH (Figure 4). The lability of the covalent linkage between RepB and the DNA has been hypothesized to play a critical role in releasing the inactive hexameric protein at the termination step, thus preventing recycling of RepB and continuous rounds of replication of the plasmid leading strand (Ruiz-Masó et al., 2015; Bordanaba-Ruiseco, 2017). According to this model, stabilization of the RepB-DNA adduct could lead to recycling of the protein and subsequent uncontrolled synthesis of the leading strand, a process that might counteract partially the impairment of the endonuclease activity at low pH. Based on the results of PCN in the host cells grown at the lowest pHs (Figures 5B,C), it does not seem however that RepB recycling, if occurs, has significant outcomes for the plasmid replication. Persistence of the covalent adduct might be a required but not sufficient condition for continuous replication using the same plasmid DNA template molecule.

Impaired RepB-mediated cleavage could result in a decreased frequency of the *in vivo* replicative events per RepB molecule, which would in turn entail a reduction in the intracellular concentration of pMV158 along with an increase in the protein to plasmid molar ratio. This is, in fact, what is observed in the lactococcal host cells grown in the absence of Tc at the lowest pH. Namely, it can be seen that at pH 4.5: 1) the PCN per chromosome equivalent (and hence the plasmid concentration) decreases by ∼50% as compared with that at pH 7.0 (Figure 5C), and 2) the ratio between the concentration of RepB and the PCN is ∼2.4-fold higher than that at pH 7.0 (Figure 6B). The decrease in the pMV158 copy number at low pH occurs even though a significant host growth slowdown, such as that observed in the most acidic medium, usually entails an increase in plasmid concentration or PCN per chromosome equivalent (Engberg and Nordström, 1975; Klumpp, 2011; van Mastrigt et al., 2018). It thus seems that the diminished pMV158 copy number observed within the host cells grown in M17’ medium adjusted to pH 4.5 is directly accounted for by the low intracellular pH. With regard to the concentration of Rep initiator molecules relative to the plasmid concentration, it has been previously reported for staphylococcal RCR plasmid pT181 (wt and various copy-number mutants) that there is approximately one RepC dimer per plasmid copy (Jin et al., 1997). We have also estimated before that in the streptococcal host (*S. pneumoniae* 708) one RepB hexamer exists per pMV158 molecule, indicating that each hexameric RepB drives one plasmid replication event (Ruiz-Masó et al., to be published elsewhere). We assume that such an efficient utilization of RepB would also occur in the lactococcal host cells grown at pH 7.0, which was taken as the reference for estimating the RepB to plasmid molar ratio at pH values of 5.0 and 4.5 (Figure 6B). This ratio is only significantly increased at pH 4.5, suggesting a less efficient utilization of RepB, with each replication event potentially requiring 2 or 3 RepB molecules in the lactococcal host cells grown at the lowest pH. However, the plasmid segregational stability points to the occurrence of regulated replication even at the lowest pH (Figure 5A).

It should be taken into account that acid-base homeostasis implies a pH gradient between the cytoplasm and the culture medium; the more extreme the pH, the greater the gradient. The pH homeostasis of *L. lactis* MG1363 in acid stress conditions has been studied and shown to entail a pH gradient value ranging from 0.7 (at outer pH 6.6) to 1.2 (at outer pH 4.4), so that the internal pH of the lactococcal cells grown in M17’ adjusted to pH 4.5 would be ∼5.6, which is the minimal internal pH value that enables the bacterial growth (Mercade et al., 2000). Hence, the pH homeostasis most likely accounts for the rather moderate reduction in PCN and plasmid concentration observed in the lactococcal host cells grown in medium at pH 4.5, which does not result in plasmid loss for at least 20 generations (Figure 5A).

The present work constitutes the first *in vitro* and *in vivo* global analysis of the potential effects of the acidity on the replication of a RCR plasmid. Previously, the effect of the culture medium pH on the PCN of RCR lactococcal plasmid pLd3, which belongs to a plasmid replicon family different from pMV158, had been investigated. No statistically significant decrease of the pLd3 PCN was found in the lactococcal cells grown at pH 5.5 (the only acidic condition investigated) compared with those grown at pH 7.0 (van Mastrigt et al., 2018). It should be highlighted nonetheless that the reduction in the pMV158 PCN in the lactococcal host is only observed at external pH values lower than 5.5, to which a cytoplasmic pH of 6.2–6.3 would be expected (Figure 5B; van Mastrigt et al., 2018)**.**

This study is also pioneer in the analysis of the pH-dependence of the different reactions, all of them involving nucleophilic attacks, catalyzed by members of the HUH endonuclease superfamily (Chandler et al., 2013). The results obtained in the present work show that the RepB-mediated DNA cleavage and the adduct hydrolysis, but not the strand-transfer reaction, are pH-dependent and greatly impaired in acidic conditions (Figures 2–4). The lability of the protein-DNA covalent linkage seems to be anyhow a singularity of the pMV158-encoded RepB initiator protein, as it has not been reported for the covalent adducts generated upon DNA cleavage by other HUH endonucleases. The lability of the protein-DNA covalent linkage has also been reported for the filamentous phage gpII RCR initiator protein, which, like the Rep proteins encoded by the pT181-family plasmids, lacks the HUH motif involved in binding of the divalent metal cation at the active site and hence does not belongs to the HUH endonuclease superfamily (Ilyina and Koonin, 1992). In the case of the pMV158 replication system, the instability of the RepB-DNA adduct may have an important physiological role in the termination step by precluding recycling and allowing the release of the hexameric inactive form of the initiator protein. In this way, the lability of the RepB-DNA covalent linkage might make it feasible the effective control of the replication, and hence of the copy number, of pMV158 by the plasmid-encoded elements that regulate the synthesis of the initiator protein, namely transcriptional repressor CopG and antisense RNAII (del Solar et al., 1995; Ruiz-Masó et al., 2015).

## Data Availability

The raw data supporting the conclusions of this article will be made available by the authors, without undue reservation.
