# Supplementary material for: Acidic pH Decreases the Endonuclease Activity of Initiator RepB and Increases the Stability of the Covalent RepB-DNA Intermediate while Has Only a Limited Effect on the Replication of Plasmid pMV158 in Lactococcus lactis
Source: Front Mol Biosci. 2021 Mar 5;8:634461. doi: 10.3389/fmolb.2021.634461 (PMC8056398; doi:10.3389/fmolb.2021.634461)
Supplement: Supplementary file 1 [file table1.docx]

Supplementary Material

Acidic pH Decreases the Endonuclease Activity of Initiator RepB and Increases the Stability of the Covalent RepB-DNA Intermediate while Has Only a Limited Effect on the Replication of Plasmid pMV158 in *Lactococcus lactis*

***Rafael Valdelvira, Lorena Bordanaba-Ruiseco, Cristina Martín-Huestamendía, José Ángel Ruiz-Masó* and Gloria del Solar****

Department of Microbial and Plant Biotechnology, Centro de Investigaciones Biológicas, Consejo Superior de Investigaciones Científicas, Madrid, Spain

*** Correspondence:**José Ángel Ruiz-Masó
[jaruizmaso@cib.csic.es](mailto:jaruizmaso@cib.csic.es)

Gloria del Solar

[gdelsolar@cib.csic.es](mailto:gdelsolar@cib.csic.es)


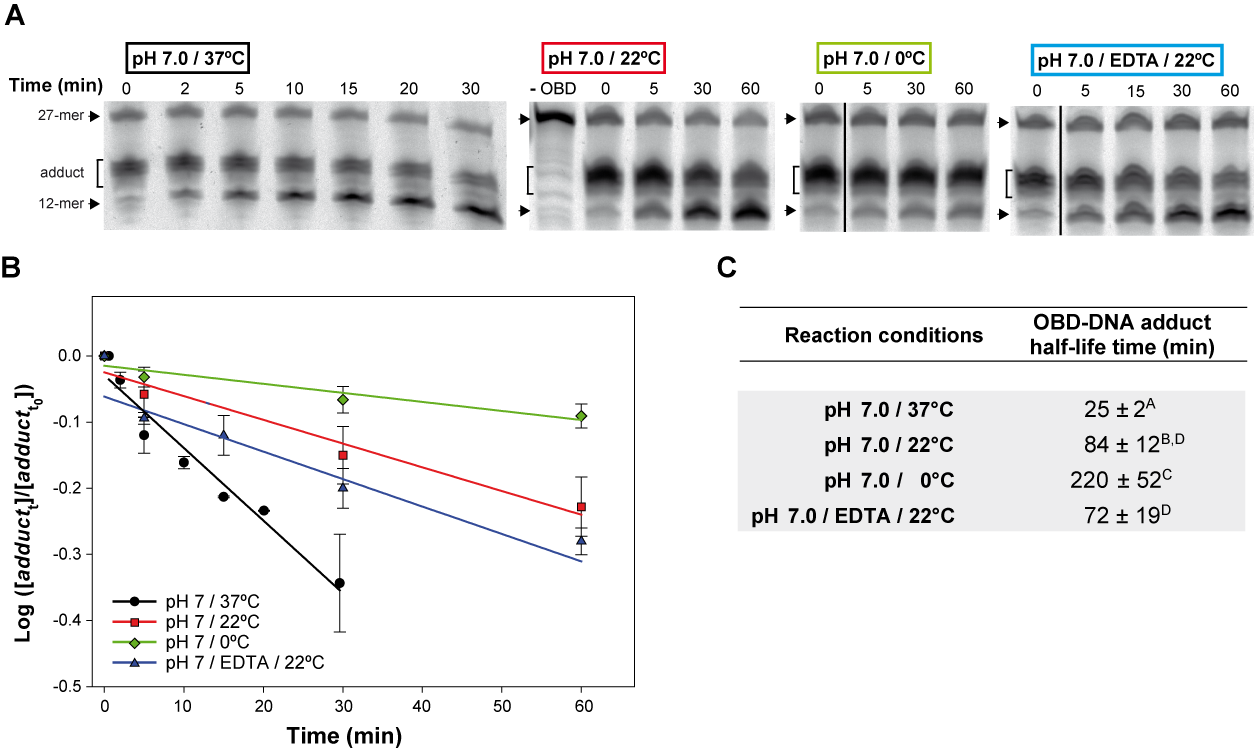


**Figure S1. OBD-DNA adduct stability at different temperatures and in the presence of EDTA. A)** Gels showing the effect of temperature and depletion of divalent cations on the time course of the adduct-hydrolysis reaction. The reaction mixtures contained a 1.5:1 molar ratio of OBD to the 3'-fluorescently labeled 27-mer oligo substrate and were prepared in buffer 7. After 2 min of incubation at 25°C, the endonuclease reaction was diluted 30 times with the same buffer equilibrated to the reaction temperature analyzed (i.e., 0°C, 22°C and 37°C). The effect of the depletion of Mn^2+^ ions was analyzed by treating the diluted reaction with 20 mM EDTA and incubating the mixture at 22°C. In all cases, several aliquots were taken at intervals and analyzed in 20% PAA-urea gels. A control lane (-OBD) showing the migration of the fluorescent 27-mer substrate is included. The dividing line indicates grouping of different parts of the same gel. **B**) Kinetics of the hydrolysis of the OBD-DNA adduct at the three temperatures analyzed and at 22°C in the presence of EDTA. The logarithm of the ratio between the fraction of adduct at each of the time intervals analyzed (*adduct*_t_) and the initial fraction (*adduct*_t0_) was plotted against time, and the experimental data were fitted to Equation (2) by linear regression. The OBD-DNA adduct half-life time was calculated from the slope of the regression curve according to Equation (3). Plots display the adduct decay curves obtained from five independent experiments for each condition, with the symbols and vertical bars representing the mean and standard deviation, respectively. **C)** Table showing the adduct half-life time calculated from five independent experiments performed under the indicated reaction conditions. The values are expressed as mean ± SD. Values with different superscript letters were significantly (P < 0.05) different.
